# Report of a Case of Absence of the Uterus, Fallopian Tubes and Ovaries, Associated with Malformation of the Vagina and Urethra

**Published:** 1889-04

**Authors:** R. H. L. Bibb

**Affiliations:** Saltillo, Mexico


### REPORT OF A CASE OF ABSENCE OF THE UTERUS, FALLOPIAN TUBES AND OVARIES, ASSOCIATED WITH MALFORMATION OF THE VAGINA AND URETHRA.

BY R. H. L. BIBB, M. D., SALTILLO, MEXICO.

[For Daniel's Texas Medical Journal.]

MRS. R——, aged twenty-one, a strong, healthy looking, finely developed Mexican woman, consulted me in 1883, concerning the condition of her generative organs. She stated that she had always enjoyed the most perfect health; that she had been married three years; had never menstruated; had never experienced any sexual desire, or sexual enjoyment ; that after her marriage, her husband had not been able to copulate with her, until—to use her own language—"the door had been opened by a surgical operation;" that since the operation the sexual act was only occasionally complete, and that at such times it was always attended with a greater or lesser flow of water, with more or less of pain, and invariably followed by a day or two of vesical irritability, partial incontinence and discharge of a little clotted blood. She denied ever to have experienced anything like a monthly molumen, or to have had either vicarious hemorrhages, or other discharges.

The patient being chloroformed—at her earnest solicitation, and it may not be out of place to state that the entire examination was conducted under the influence of that drug—it was noted that the chest was broad, full and prominent; the mammary glands were small and flaccid; the nipples small, almost rudimental in character; waists tapering, pelvis and hips broad and roomy; legs, round and tapering; hands and feet small and effeminate; facial expression, feminine; head covered with a luxuriant growth of hair. In short, the patient's physique represented the strong, healthy, robust female type.

By inspection, the mons veneris was found well covered with hair; the labia majora and minora, the clitoris and the perineum, normally developed; large and prominent remains of the meatus urethræ.

On introducing my index finger into the vagina, I found that canal to be one inch in depth, somewhat conoidal in shape, with its apex terminating in the meatus urethræ, the latter permitting my finger to pass on into the bladder, through an urethra about a third of an inch long.

I next instituted search for the uterus by passing a long curved blunt steel sound into the bladder and my right index finger into the rectum. In this way, search was made in every direction; upwards and downwards in the median line, to the right and to the left, but nothing could be found representing the slightest trace of that organ, even in its most rudimentary form—the point of the instrument touching my finger, whenever the two were approximated; nor could I, with the left index finger in the bladder, the right in the rectum, with strong pressure, by an assistant behind the pubis, over the hypogastric, and in the right and left inquinal regions, after a very careful examination, detect any trace whatever, of either the uterus, fallopian tubes or ovaries. Pushing my investigation a little further, I again examined for the left ovary and fallopian tube with the index finger of my left hand in the rectum, while I made counter pressure with my right over the left inguinal region, and deep down into the pelvis, while for those of the right side, with the right index finger in the bladder, but with like negative results.

This lady and her husband, desiring a refutation or a confirmation of my diagnosis and prognosis, especially with reference to child-bearing, about a year ago, placed herself under the care of Dr. Smith, of this city, a gynecologist of large experience and of no mean ability. The Doctor subjected her to several extended and searching examinations, and finally agreed with me as to the complete absence of the uterus, the fallopian tubes and ovaries, and to the existence of the malformations narrated above.

In a conversation with Dr. Porth, a well learned German physician of this city, concerning Mrs. R.'s condition, and subsequent to the examination herein detailed, he, who made the operation—"opened the door," I learned that some few months after her marriage, he was called upon to remove some barrier to the sexual act; that he found a thickened, rigid, unyielding hymen circularis which he divided by a crucial incision, but, as his examination extended no farther, he was unable to say whether my diagnosis of the case was correct or not.

The rare occurrence of such cases as is the subject of this report, shall constitute my apology, if any is needed, for presenting it to the profession. True, none of the conditions, if existing alone, would be of any special interest, but taken as a whole,—the unyielding hymen removed by Dr. Porth, the malformation of the vagina and of the urethra, the latter occasionally taking the place of the former in the act of copulation, forundoubtedly the phenomena described by Mrs. R., as attending and following coition, are produced by the male organ entering the bladder, the absence of the uterus, the fallopian tubes and of the ovaries—they make up a case that is exceedingly rare, and one which in my humble judgment, will not be without interest to some, especially when it is remembered that many eminent gynecologist deny that a positive diagnosis of complete absence of the uterus can be made during life.

